# Therapeutic Value of Budesonide Nasal Irrigation for Postoperative Maintenance of Chronic Rhinosinusitis: A Real-World Study

**DOI:** 10.3390/jcm15197617

**Published:** 2026-10-01

**Authors:** Soo Yeon Kim, Jin-Young Min

**Affiliations:** Department of Otorhinolaryngology—Head and Neck Surgery, Kyung Hee University Hospital, Kyung Hee University College of Medicine, Seoul 02447, Republic of Korea

**Keywords:** chronic rhinosinusitis, budesonide nasal irrigation, endoscopic sinus surgery, topical corticosteroids, postoperative care

## Abstract

**Objectives:** Budesonide irrigation (BI) has emerged as an effective postoperative topical therapy for chronic rhinosinusitis (CRS), providing improved drug delivery compared with conventional intranasal corticosteroid sprays. However, evidence regarding its role as a maintenance strategy during the critical postoperative period remains limited. This study aimed to evaluate the clinical efficacy of BI compared with saline irrigation (SI) in patients with persistent symptoms following functional endoscopic sinus surgery (FESS) and to determine whether treatment effects differed according to CRS inflammatory endotypes. **Methods:** This retrospective cohort study included 121 patients with CRS who underwent FESS between 2021 and 2024. Patients with persistent symptoms 3 months post-operation were treated with either continued SI (*n* = 54) or BI (*n* = 67) for at least 6 months. Clinical outcomes included changes in SNOT-22 scores, modified Lund–Kennedy endoscopic scores (mLKS), olfactory function assessed by KVSS II, oral corticosteroid (CS) use, and disease control status. Subgroup analyses were conducted for eosinophilic CRS (ECRS) and non-eosinophilic CRS (NECRS). **Results:** The BI group showed significantly greater improvement in total SNOT-22 scores than the SI group (*p* < 0.0001), particularly in rhinological symptoms. Endoscopic outcomes also favored BI, with greater reductions in total mLKS and mucosal edema scores. These benefits were observed in both ECRS and NECRS, although objective endoscopic improvements were more pronounced in ECRS. Patients receiving BI required fewer courses of oral CS (18.42% vs. 52.38%, *p* = 0.01) and demonstrated better disease control (*p* = 0.03). However, no significant difference was observed in olfactory function. **Conclusions:** BI provides superior symptom improvement, better endoscopic healing, and reduced oral CS use compared with SI during postoperative CRS maintenance, supporting its use in both ECRS and non-ECRS subgroups.

## 1. Introduction

Chronic rhinosinusitis (CRS) is a chronic inflammatory disorder of the paranasal sinuses affecting approximately 5% to 12% of the global population, leading to a significant reduction in quality of life and substantial socioeconomic costs [1,2]. Traditionally phenotyped into CRS with nasal polyps (CRSwNP) and without polyps (CRSsNP), recent guidelines, such as EPOS 2020, have shifted toward an endotype-based classification, emphasizing the critical role of Type 2 (T2) inflammation [3,4,5,6]. Patients with eosinophilic CRS (ECRS), often associated with comorbid asthma, face a particularly high risk of mucosal edema and polyp recurrence even after successful functional endoscopic sinus surgery (FESS) [4,5]. Despite the introduction of personalized therapeutic strategies based on endotypes, managing patients with persistent symptoms—classified as having difficult-to-treat rhinosinusitis—remains a formidable clinical challenge [5].

FESS remains the gold standard for the surgical management of CRS; however, postoperative management is crucial to maintain sinus patency and suppress recurrent inflammation. While intranasal corticosteroid (INCS) sprays are the standard first-line treatment, they are often limited by inadequate delivery and poor penetration into the complex anatomy of sinus cavities that have been operated on, particularly in cases with persistent mucosal swelling [7]. To overcome these limitations, high-volume, positive-pressure steroid nasal irrigation has emerged as a superior delivery system, significantly improving drug distribution throughout the paranasal sinuses. Specifically, budesonide nasal irrigation has been increasingly utilized off-label for its potent local anti-inflammatory effects [8,9,10]. Although not yet FDA-approved for this indication, numerous studies have demonstrated its safety profile, with no serious short-term or long-term adverse effects reported [11,12]. Furthermore, budesonide nasal irrigation following FESS has been shown to be more effective than INCS alone in improving objective measures, such as the Lund–Mackay score (LMS) and Lund–Kennedy score (LKS), as well as subjective nasal obstruction symptoms [8,9,10]. Recent evidence also suggests that long-term budesonide nasal irrigation use in patients with CRSwNP and asthma leads to significant symptomatic improvement and a reduced reliance on systemic corticosteroids and antibiotics [13].

Crucially, the anti-inflammatory reach of corticosteroids in the sinonasal mucosa is not limited to eosinophilic pathways. While steroids are the cornerstone of T2-inflammation management, they also exert pleiotropic effects on non-eosinophilic (Type 1/Type 3) pathways by inhibiting pro-inflammatory cytokines (e.g., IL-1, IL-6, and TNF-α) and modulating neutrophil-related inflammation [14]. This broad-spectrum efficacy makes budesonide nasal irrigation a potential candidate for treating various CRS endotypes, yet there remains a lack of standardized evidence regarding its efficacy during the critical one-year postoperative window, a period when recurrence frequently concerns both patients and clinicians.

When standard postoperative outcomes are poor, clinicians often face the dilemma of escalating treatment, such as with systemic corticosteroids or biologics. However, these options are frequently limited by high costs and/or potential systemic side effects. Therefore, exploring budesonide nasal irrigation as a cost-effective, non-surgical alternative is highly warranted. This study aimed to evaluate the efficacy of budesonide irrigation (BI) compared with saline irrigation (SI) in patients with partly controlled or uncontrolled CRS following FESS, focusing on subjective symptoms, objective endoscopic healing, and its potential to reduce the need for systemic rescue medications in both ECRS and non-ECRS subgroups. Furthermore, this study sought to determine whether the therapeutic efficacy of BI varies according to ECRS and non-ECRS subgroups of CRS.

## 2. Materials and Methods

This retrospective cohort study included patients diagnosed with CRS according to the EPOS 2020 criteria who underwent FESS at our institution between January 2021 and February 2024. Following surgery, all patients received a standardized medical management regimen for the initial postoperative phase, consisting of a 2-week course of systemic antibiotics, INCS sprays, antihistamines, mucolytics, and nasal SI.

To evaluate the efficacy of long-term topical therapy in difficult-to-treat cases, we specifically identified a subgroup of patients who reported persistent symptoms at the 3-month postoperative mark. These patients were subsequently transitioned to a specialized topical treatment protocol (either BI or continued SI) for a minimum of 6 months. To assess long-term outcomes and the prevention of early recurrence, only patients with a minimum follow-up period of 12 months post-surgery were included. The primary objective was to compare the degree of improvement in subjective symptoms and objective endoscopic findings between the treatment groups while also investigating whether these outcomes varied significantly according to the underlying inflammatory endotype.

At three months post-operation, patients whose Sino-Nasal Outcome Test-22 (SNOT-22) score remained greater than 20 were considered to have persistent symptoms and were subsequently managed with continued nasal irrigation therapy [15]. Among these patients, those who continued irrigation therapy for at least 6 months were included in the analysis. Patients were classified into two groups according to the irrigation regimen used during the follow-up period. Treatment selection was not based on a fixed protocol but was determined during routine clinical practice according to the treating clinician’s clinical judgment and patient preference. The SI group continued standard nasal saline irrigation, whereas the BI group received budesonide-mixed saline irrigation. The budesonide irrigation solution consisted of 240 mL of normal saline mixed with 0.5 mg/2 mL of budesonide suspension and was administered once or twice daily according to routine clinical practice. Both groups continued to use INCS sprays throughout the study period. For subgroup analysis, patients were further stratified according to CRS endotype into ECRS and NECRS based on established diagnostic criteria. ECRS was defined as the presence of either a peripheral blood eosinophil count ≥ 150 cells/µL or tissue eosinophil infiltration ≥ 70 cells per high-power field [16,17]. Treatment outcomes were then compared between the irrigation groups within each subgroup. Patients were excluded if they received biologics including dupilumab or omalizumab, underwent allergen immunotherapy including subcutaneous or sublingual immunotherapy, required additional postoperative systemic antibiotics such as doxycycline, or had a history of revision endoscopic sinus surgery. Only patients who met these criteria and had sufficient follow-up data were included in the final analysis.

Subjective symptom outcomes were evaluated using the total and individual item scores of the SNOT-22 questionnaire. Changes in symptom scores from baseline to follow-up were compared between the SI and BI groups after at least 6 months of irrigation therapy. In addition to total score changes, domain-specific analyses were performed to evaluate rhinologic, otologic/facial, sleep, and emotional symptom domains (Table 1) [18].

Objective endoscopic findings were assessed using the modified Lund–Kennedy Score (mLKS), including component scores for nasal polyps, mucosal edema, and nasal discharge, each graded on a scale of 0 to 2. Changes in the mLKS from baseline were analyzed to evaluate the degree of mucosal inflammation and endoscopic disease activity during follow-up [19,20].

Olfactory function was assessed using the Korean Version of the Sniffin’ Sticks Test II (KVSS II). The total KVSS II score was compared between baseline and follow-up evaluations in patients who underwent both pre- and post-treatment olfactory testing. In addition, the requirement for rescue medication during the follow-up period was recorded by assessing the use of systemic corticosteroids. Overall disease control status was evaluated according to the EPOS 2020 criteria and categorized as controlled, partly controlled, or uncontrolled [2].

Changes in clinical outcomes, including SNOT-22 scores, mLKS, and KVSS II scores, were calculated from baseline values and compared between the BI and SI groups. Subgroup analyses were conducted for eosinophilic and non-eosinophilic CRS populations. Differences in the proportion of patients requiring oral corticosteroids (CS) and the distribution of disease control status were analyzed. All participants provided informed consent to participate, and the study was approved by the Institutional Review Board of the participating institution (No. 2026-07-019).

Statistical analyses were performed using GraphPad Prism software (version 10.6.1; GraphPad Software, San Diego, CA, USA). Continuous variables were expressed as mean ± standard deviation (SD), whereas categorical variables were presented as frequencies and percentages. Differences between groups were analyzed using the Mann–Whitney U test, independent *t*-test, chi-square tests or Fisher’s exact tests for categorical variables. A two-tailed *p* value of less than 0.05 was considered statistically significant.

## 3. Results

Baseline demographic and clinical characteristics of the study population are summarized in Table 2. A total of 121 patients were included in the analysis, of whom 54 received saline irrigation (SI group) and 67 received budesonide irrigation (BI group). Baseline comorbidities, CRS phenotype and endotype distributions, and laboratory parameters did not differ significantly between the two groups.

### 3.1. Improvements in Subjective Symptoms (SNOT-22)

Patient-reported symptom outcomes were assessed by comparing changes in SNOT-22 scores, where the BI group demonstrated a greater reduction in total SNOT-22 score compared with the SI group (Figure 1). The mean reduction in the total SNOT-22 score from baseline was significantly greater in the BI group compared with the SI group (−16.45 ± 11.28 vs. −7.93 ± 7.97 respectively, *p* < 0.0001), a trend that remained consistent regardless of the underlying endotype, including both ECRS (−17.21 ± 10.53 vs. −7.22 ± 8.80 respectively, *p* = 0.0003) and NECRS subgroups (−15.39 ± 12.37 vs. −8.45 ± 7.41 respectively, *p* = 0.03; Figure 1A). These findings indicate broad-based symptomatic improvement associated with topical corticosteroid irrigation.

As shown in Figure 1B, domain-specific analysis revealed consistent patterns favoring BI across rhinological symptoms in total patients (−12.00 ± 7.34 vs. −3.50 ± 5.69, respectively, *p* < 0.0001), ECRS patients (−12.00 ± 7.47 vs. −3.22 ± 7.10, respectively, *p* < 0.0001), and NECRS patients (−10.25 ± 4.77 vs. −3.71 ± 4.49, respectively, *p* < 0.0001). However, unlike rhinological symptoms, there were no statistically significant differences between the two groups in the otologic/facial (Figure 1C), sleep (Figure 1D), or emotional domains (Figure 1E, all *p* > 0.05).

### 3.2. Objective Endoscopic Findings (mLKS)

Changes in mLKS from baseline are shown in Figure 2. In terms of the total mLKS (Panel A), the BI group demonstrated a significantly greater reduction compared with the SI group in the overall cohort (−2.91 ± 2.13 vs. −1.26 ± 2.08, respectively, *p* < 0.001) and in the ECRS subgroup (−3.26 ± 2.11 vs. −1.17 ± 2.25, respectively, *p* < 0.01). In the non-ECRS subgroup, a greater reduction was also observed in the BI group compared to the SI group; however, this difference did not reach statistical significance (−2.48 ± 2.11 vs. −1.35 ± 1.95 respectively, *p* = 0.06).

For the polyp score (Panel B), there were no statistically significant differences between the two groups in the total population or in either the ECRS or non-ECRS subgroups (all, *p* > 0.05). In the edema score (Panel C), the BI group showed significantly greater improvement than the SI group in the overall cohort (−1.58 ± 1.36 vs. −0.50 ± 1.06, respectively, *p* < 0.0001) and in the ECRS subgroup (−1.92 ± 1.31 vs. −0.43 ± 1.16, respectively, *p* < 0.0001). In the non-ECRS subgroup, a greater reduction was observed in the BI group, but this difference did not reach statistical significance (−1.11 ± 1.32 vs. −0.55 ± 0.99 respectively, *p* = 0.07). Similarly, in the discharge score (Panel D), the BI group demonstrated numerically greater reductions compared with the SI group across all groups. However, these differences were not statistically significant in the total cohort (−1.40 ± 1.45 vs. −0.93 ± 1.47, respectively, *p* = 0.07) or in the non-ECRS subgroup (−1.61 ± 1.34 vs. −0.97 ± 1.38, respectively, *p* = 0.08), and no significant intergroup difference was observed in the ECRS subgroup (−1.26 ± 1.52 vs. −0.87 ± 1.60, respectively, *p* = 0.33).

Overall, the BI group showed significantly greater improvements in the total mLKS and component analysis demonstrated superior improvement in mucosal edema, indicating that budesonide irrigation contributed to meaningful reductions in mucosal inflammation and disease activity observable on endoscopy.

### 3.3. Olfactory Function

Olfactory function changes were assessed using KVSS II total scores (Figure 3). Both treatment groups demonstrated postoperative improvement in olfactory performance. Although the difference did not reach statistical significance (*p* = 0.09), the increase in the mean KVSS II score was greater in the BI group (5.63 ± 4.88, *n* = 19) than in the SI group (2.43 ± 3.69, *n* = 14). This trend suggests a potentially beneficial effect of BI on olfactory recovery, possibly mediated through improved control of mucosal inflammation and restoration of nasal airflow. However, the number of patients who underwent olfactory testing both before and after treatment was limited; therefore, further studies with larger sample sizes are needed to confirm these findings.

### 3.4. Oral Corticosteroid (CS) Requirements and Clinical Disease Control Levels

The use of BI also impacted the need for rescue medication (Figure 4A). After we excluded patients with comorbidities precluding systemic corticosteroid use, such as diabetes mellitus or glaucoma, a significantly higher proportion of patients in the SI group (*n* = 11, 52.38%) required oral CS prescriptions compared with those in the BI group (*n* = 7, 18.42%; *p* = 0.01), indicating a reduced need for systemic rescue therapy with BI.

Finally, overall disease control status was evaluated according to clinical criteria (Figure 4B). A higher proportion of patients achieved ‘Controlled’ status in the BI group (*n* = 13, 34.22%) compared to the SI group (*n* = 2, 9.52%). Conversely, the SI group had a higher frequency of patients categorized as ‘Uncontrolled’ (*n* = 9, 42.86%) or ‘Partly Controlled’ (*n* = 10, 47.62%). This shift toward better clinical outcomes in the BI group was statistically significant (*p* = 0.03), underscoring the therapeutic advantage of adding budesonide to the irrigation regimen.

## 4. Discussion

This study demonstrates that employing BI as a postoperative maintenance strategy provides superior clinical benefits compared with conventional SI during the critical 6-to-12-month postoperative period. The therapeutic advantages of BI were consistently observed across multiple clinical domains, including patient-reported symptom improvement, enhanced endoscopic mucosal healing, and reduced reliance on systemic rescue medications. Importantly, these benefits were observed across both ECRS and NECRS subgroups, suggesting that high-volume corticosteroid irrigation may provide broad anti-inflammatory effects across heterogeneous CRS phenotypes.

Corticosteroids have traditionally been considered most effective in T2-high inflammatory conditions such as ECRS because of their potent ability to induce eosinophil apoptosis and suppress type 2 inflammatory signaling pathways [2,14,21]. Our findings further reinforce this concept. In the ECRS subgroup, BI resulted in a markedly greater reduction in total SNOT-22 scores compared with SI (−17.21 ± 10.53 vs. −7.22 ± 8.80, *p* = 0.0003), with the most pronounced improvements observed in the rhinological symptom domain. These results highlight the effectiveness of high-volume irrigation in delivering corticosteroids directly to inflamed sinonasal mucosa. Because ECRS is characterized by severe mucosal edema and a high risk of postoperative recurrence, effective topical steroid delivery plays a crucial role in maintaining disease control following surgery.

Objective endoscopic findings further supported the clinical benefits of BI. The BI group showed significantly greater improvement in mucosal edema compared with the SI group (−1.92 ± 1.31 vs. −0.43 ± 1.16, *p* = 0.0002), which represents an important indicator of postoperative inflammatory control and a key predictor of early disease recurrence. Evaluation using the mLKS also demonstrated significantly greater reductions in total scores in the BI group. Component analysis revealed superior improvement in polyp size, mucosal edema, and nasal discharge, indicating that BI effectively reduced mucosal inflammation and disease activity observable on endoscopy. In the ECRS subgroup, the beneficial effect of BI on endoscopic findings remained pronounced, with greater decreases in both total and component mLKS values compared with SI. These results further reinforce the known responsiveness of eosinophilic inflammatory disease to corticosteroid therapy [2,3,16,22].

In addition to improvements in symptoms and endoscopic findings, healthcare utilization patterns also differed between the treatment groups. Analysis of oral CS prescriptions during the follow-up period revealed that a significantly greater proportion of patients in the SI group required systemic corticosteroid therapy compared with those receiving BI (Figure 4A). Furthermore, overall disease control status was evaluated using predefined clinical control criteria (Figure 4B) [2]. Distribution analysis demonstrated a higher proportion of patients categorized as “Controlled” and a corresponding reduction in those classified as “Uncontrolled” in the BI group compared with the SI group (*p* = 0.03). These findings suggest that the clinical benefits of BI extend beyond symptom relief and endoscopic improvement, translating into improved overall disease control and reduced need for systemic rescue therapy.

Interestingly, clinically meaningful improvement was also observed in patients with NECRS. In this subgroup, the BI group achieved significantly greater reductions in total SNOT-22 scores compared with the SI group (−15.39 ± 12.37 vs. −8.45 ± 7.41, *p* = 0.03). Notably, the observed benefit of BI was more consistent for patient-reported symptom outcomes, particularly SNOT-22, whereas differences in objective endoscopic outcomes were less consistent, and no significant additional benefit was observed for olfactory function. Although the magnitude of endoscopic improvement in NECRS was less pronounced and did not consistently reach statistical significance across all mLKS parameters, numerical trends generally favored budesonide irrigation. This clinical benefit in non-eosinophilic disease may be explained by the pleiotropic anti-inflammatory mechanisms of corticosteroids that extend beyond eosinophilic suppression [14]. NECRS is frequently associated with Type 1 or Type 3 immune responses and neutrophilic inflammation [4]. Corticosteroids inhibit several key transcription factors involved in inflammatory signaling, particularly nuclear factor-κB (NF-κB) and activator protein-1 (AP-1), thereby suppressing the production of multiple pro-inflammatory cytokines including IL-1β, IL-6, IL-8, and TNF-α [14]. Through these mechanisms, corticosteroids can modulate neutrophil recruitment and attenuate chronic mucosal inflammation even in disease phenotypes traditionally considered less responsive to steroid therapy.

The mechanical characteristics of high-volume irrigation may further contribute to the observed therapeutic effects [5,7,8,9,10,23,24,25,26]. The positive-pressure irrigation system facilitates penetration of the medication into surgically opened sinus cavities and deeper sinus recesses, allowing more effective distribution of corticosteroids across inflamed mucosal surfaces [8]. This enhanced drug delivery may provide sufficiently high local corticosteroid concentrations to modulate inflammatory pathways and support mucosal healing throughout the sinonasal cavity [8].

The reduced need for systemic corticosteroid therapy observed in the BI group further highlights the clinical relevance of this treatment strategy. Minimizing cumulative systemic steroid exposure is a key goal in the long-term management of CRS because repeated courses of oral corticosteroids are associated with substantial adverse effects, including osteoporosis, metabolic complications, and hypothalamic–pituitary–adrenal axis suppression [27]. In healthcare environments where biologic therapies such as dupilumab remain expensive or are restricted by insurance coverage criteria, high-volume corticosteroid irrigation may represent a cost-effective and accessible maintenance option for patients with difficult-to-treat CRS.

A notable strength of this study is its focus on a clinically meaningful postoperative interval, the 6-to-12-month period following FESS. While early postoperative inflammation generally subsides within the first few months after surgery, disease recurrence frequently begins to manifest during this intermediate phase. By specifically examining this time window, our study provides important insights into the role of BI as a maintenance strategy aimed at stabilizing the postoperative sinonasal environment. In addition, the use of a standardized irrigation regimen (0.5 mg of budesonide in 240 mL of saline administered once or twice daily) provides a reproducible treatment protocol that may help address the variability in dosing strategies reported in previous studies [10,23].

Several limitations of this study should be noted. First, the retrospective design limits the ability to establish causal relationships. Second, treatment adherence was not objectively monitored. Long-term irrigation therapy requires consistent patient compliance, and variability in adherence may influence treatment outcomes. Third, although subgroup analyses were performed according to inflammatory endotype, detailed biomarker profiling was not available for all patients, which limits further investigation into the biological mechanisms underlying differential treatment responses. In addition, although the baseline characteristics were generally comparable between the groups, residual confounding cannot be completely excluded because of the retrospective and non-randomized design. Future prospective studies incorporating multivariable analyses are warranted to further validate these findings. Finally, although olfactory function showed numerically greater improvement in the BI group, the difference did not reach statistical significance, likely due to the limited number of patients who completed both baseline and follow-up olfactory testing. In terms of safety, no clinically meaningful differences in adverse events were observed between the two treatment groups during the follow-up period. These findings are consistent with previous studies reporting that BI is generally well tolerated with minimal systemic absorption. Prior investigations have demonstrated that high-volume BI does not result in clinically significant hypothalamic–pituitary–adrenal (HPA) axis suppression or systemic corticosteroid-related adverse effects when used in standard postoperative regimens. In addition, several prospective studies evaluating postoperative corticosteroid irrigation have reported favorable safety profiles with no significant increases in local complications such as epistaxis or mucosal atrophy [11,12,28]. Nevertheless, further studies with longer follow-up periods are warranted to more comprehensively evaluate the long-term safety of repeated corticosteroid irrigation in patients requiring prolonged maintenance therapy.

## 5. Conclusions

In this real-world retrospective cohort, postoperative BI was associated with greater improvement in patient-reported symptoms and selected endoscopic outcomes compared with SI, with improvements observed in both ECRS and non-ECRS subgroups. Given the observational and non-randomized design, these findings should be interpreted as associations and require confirmation in prospective randomized studies.

## Figures and Tables

**Figure 1:**
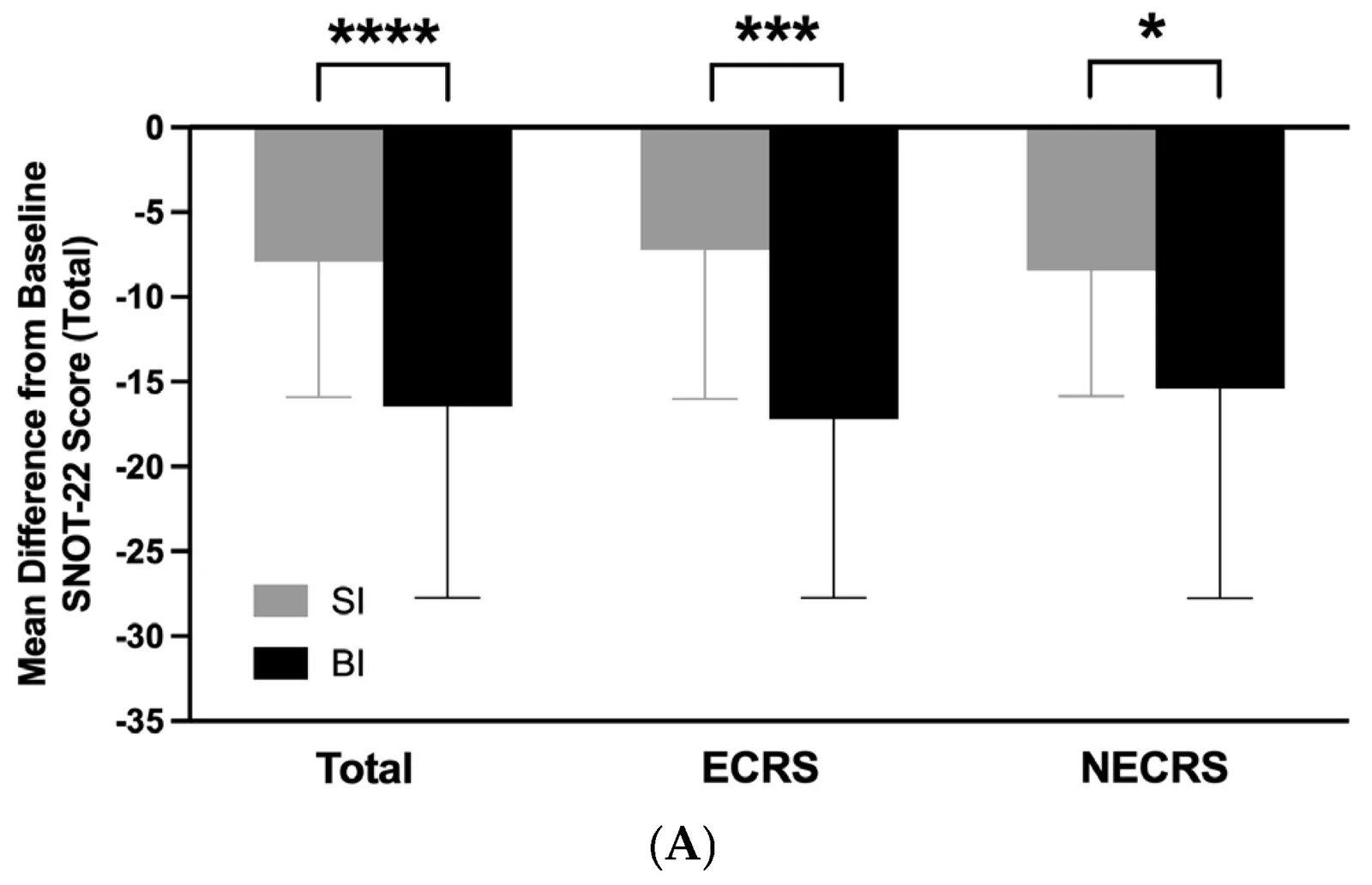

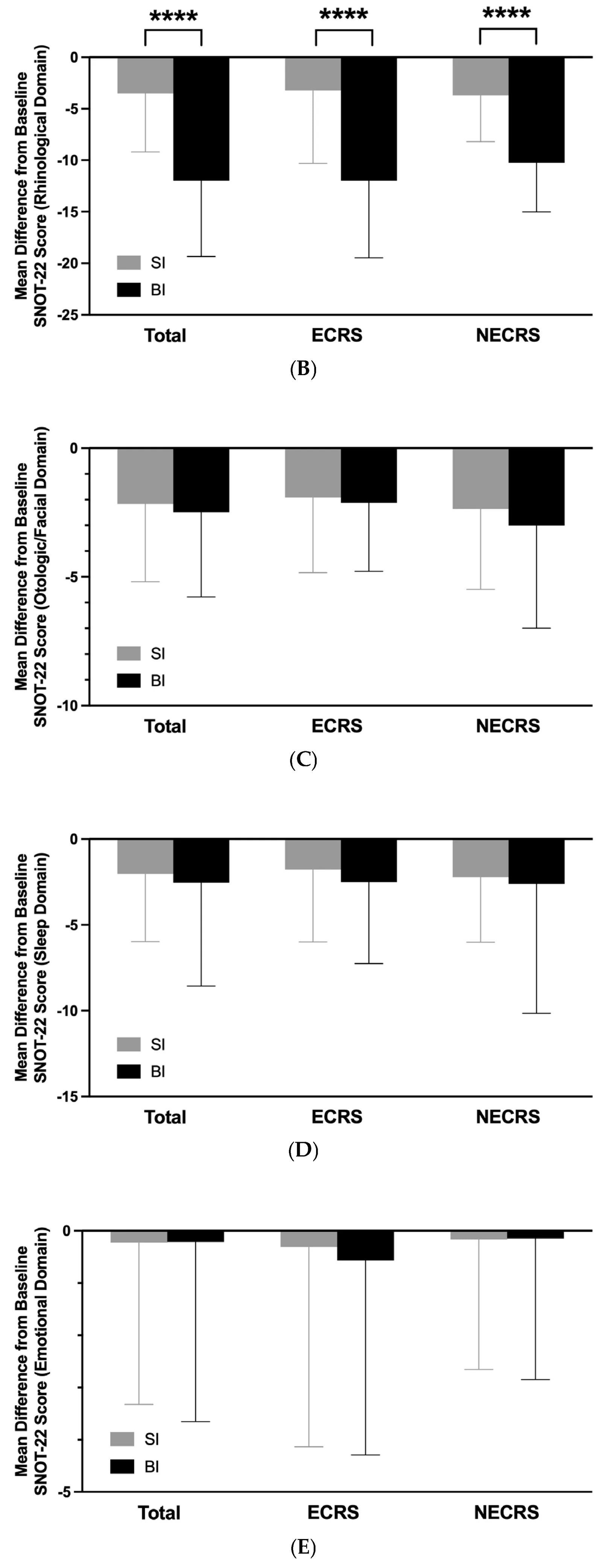
Differences in changes of SNOT-22 scores between saline irrigation (SI) and budesonide irrigation (BI) groups. Changes in SNOT-22 scores from baseline to post-treatment were calculated for each group and compared between the SI group and the BI group. (**A**) Total scores, (**B**) Rhinological domain scores, (**C**) Otologic/Facial domain scores, (**D**) Sleep domain scores, and (**E**) Emotional domain scores. Data are presented as mean ± SD. * *p* < 0.05, *** *p* < 0.001, **** *p* < 0.0001.

**Figure 2:**
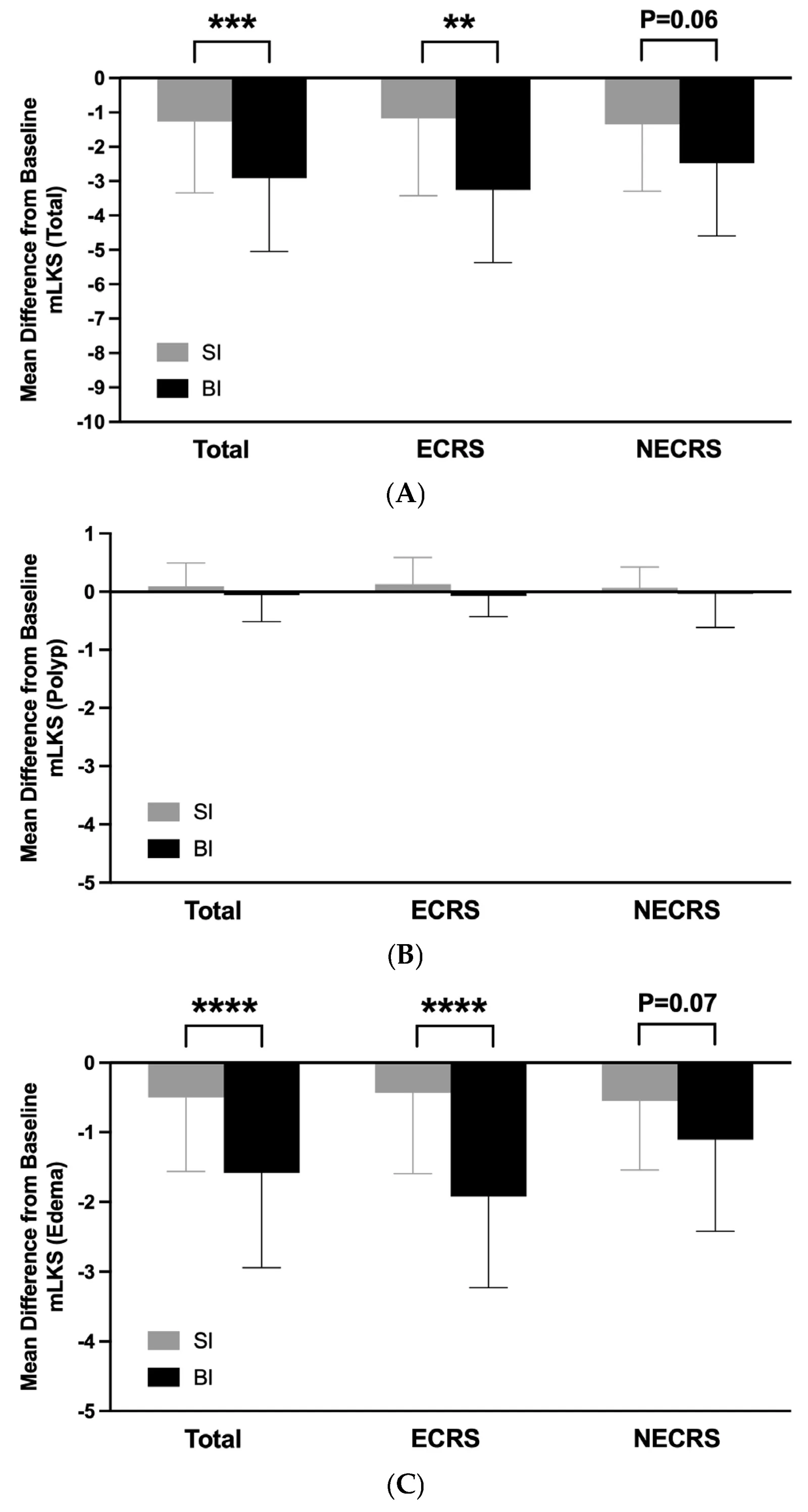

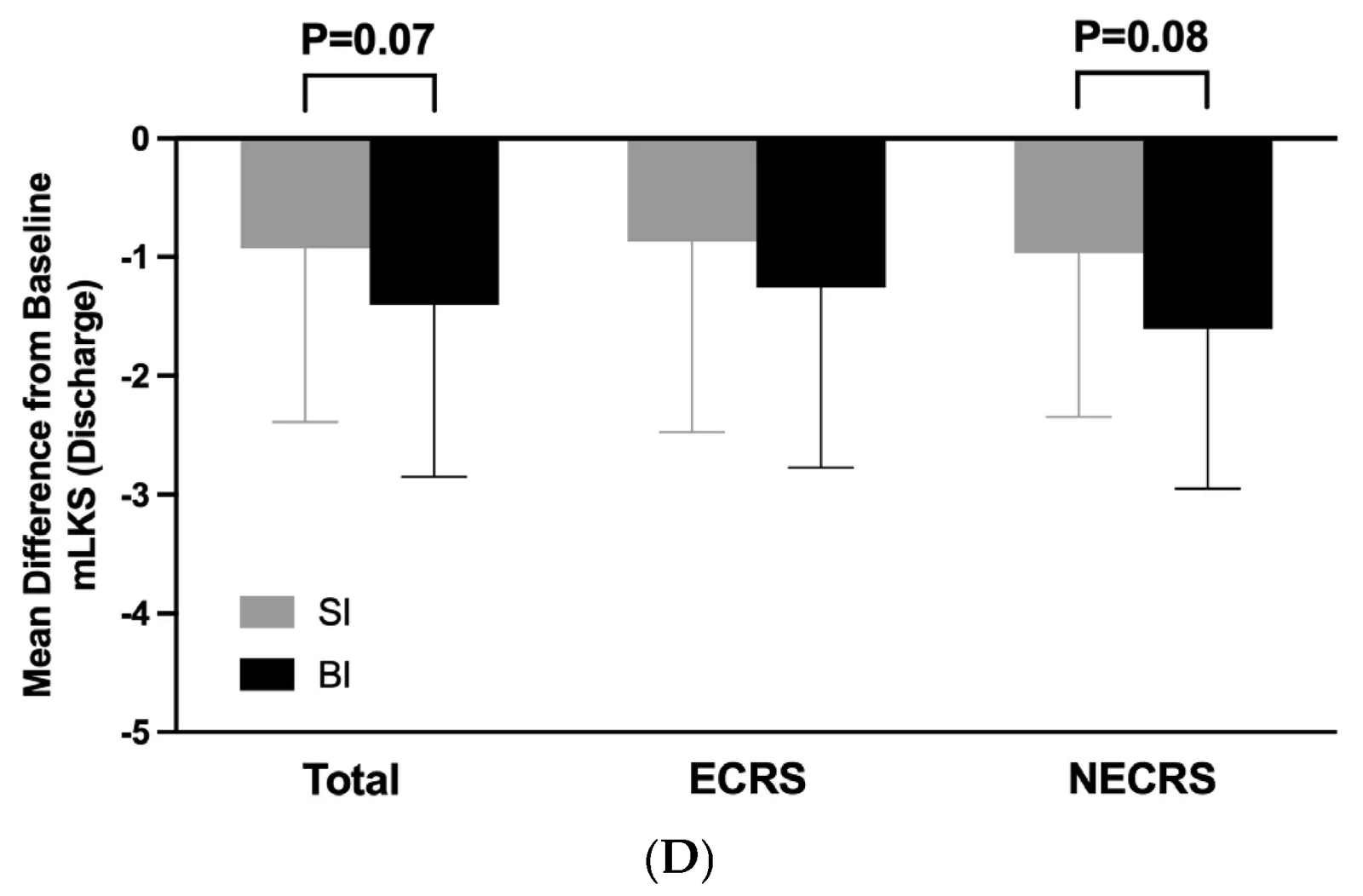
Differences in changes of modified Lund–Kennedy endoscopic scores between saline irrigation (SI) and budesonide irrigation (BI) groups. Changes in modified Lund–Kennedy endoscopic scores (mLKS) from baseline to post-treatment were calculated for each group and compared between the SI group and the BI group. (**A**) Total scores, (**B**) Polyp scores, (**C**) Edema scores, and (**D**) Discharge scores. Data are presented as mean ± SD. ** *p* < 0.01, *** *p* < 0.001, **** *p* < 0.0001.

**Figure 3:**
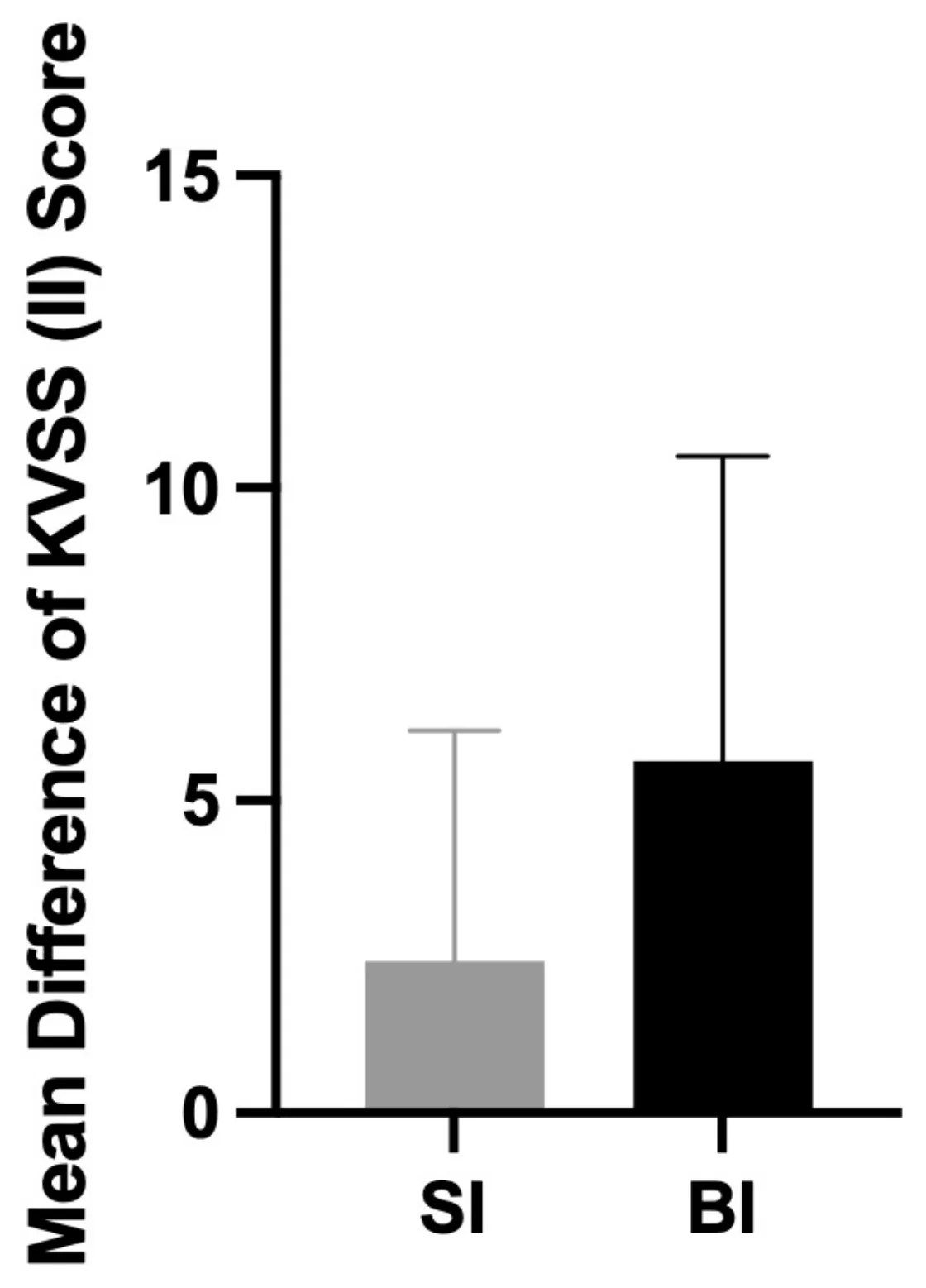
Change in olfactory function. Changes in KVSS (II) total scores from baseline to post-treatment were compared between the saline irrigation (SI) group and the budesonide irrigation (BI) group. The BI group showed a greater increase in total olfactory scores than the SI group. Data are presented as mean ± SD.

**Figure 4:**
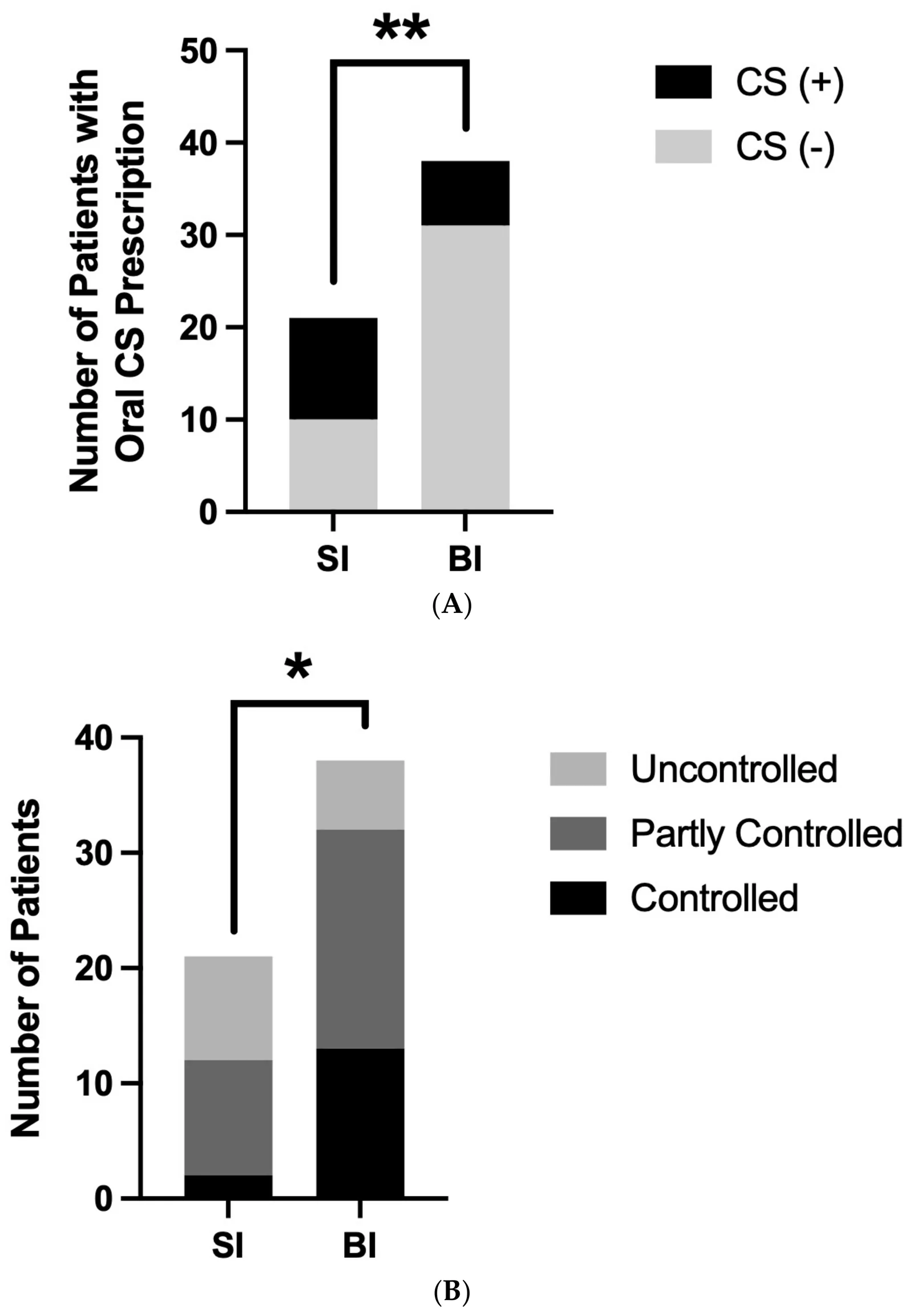
Oral corticosteroid use and disease control status during follow-up. Patients were followed for at least 6 months to 12 months after surgery while continuing nasal irrigation for at least 6 months. (**A**) Number of patients prescribed oral corticosteroids (CS+) and those not prescribed oral corticosteroids (CS−) in the saline irrigation (SI) and budesonide irrigation (BI) groups. (**B**) Distribution of disease control status (controlled, partly controlled, and uncontrolled) according to clinical control criteria. Data are presented as the number of patients. * *p* < 0.05, ** *p* < 0.01.

**Table 1:** SNOT-22 questionnaire and subdomain.

Subdomain	SNOT-22 Questionnaire Item
Rhinological domain	1. Need to “blow” your nose
	2. Sneezes
	3. “Running” nose
	4. Cough
	5. Nasal secretion going to your throat
	6. Thick secretion from your nose
	21. Difficulty to feel “smells” or “tastes”
	22. Stuffed nose
Otoloical/facial domain	7. A feeling of full or stuffed ear
	8. Dizziness or vertigo
	9. Ear ache
	10. Facial pain or pressure
Sleep domain	11. Difficulty to sleep
	12. Wake up in the middle of the night
	13. Lack of a good night of sleep
	14. Wake up tired
	15. Fatigued or tired during the day
	16. Low performance in doing daily activities
	17. Low concentration to do daily activities
	18. Frustrated, restless or irritated
Emotional domain	19. Sadness
	20. A feeling of shame

**Table 2:** Characteristics of patients.

	Total (*n* = 121)	SI (*n* = 54)	BI (*n* = 67)	*p*-Value (SI vs. BI)
Number of patients (M/F)	59:62	21:33	38:29	0.156
Age	49.8 ± 12.69	51.7 ± 11.2	47.9 ± 13.7	0.799
Allergy (Y/N/U)	39/71/10	21/31/2	18/40/8	0.397
HTN (Y/N/U)	17/71/0	7/42/0	10/57/0	0.506
Asthma (Y/N/U)	11/77/33	6/31/17	5/46/16	0.093
Smoke (Y/N/U)	23/98/0	10/44/0	13/54/0	0.104
Alcohol (Y/N/U)	71/50/0	32/22/0	39/28/0	0.124
Previous ESS (Y/N)	5/116/0	3/51/0	2/64/0	0.314
CRSwNP: CRSsNP	105:36	46:18	59:18	0.640
ECRS: NECRS	62:59	23:31	39:28	0.134
Serum Eo	284.23 ± 170.71	275.56 ± 156.87	292.91 ± 184.54	0.271
Serum ECP (µg/L)	29.87 ± 37.67	25.71 ± 41.30	34.03 ± 37.04	0.333
Serum Total IgE (IU/mL)	321.29 ± 314.08	318.05 ± 411.15	324.53 ± 217.01	0.143
Tissue Eo (Mean Eo counts/HPF)	69.34 ± 64.37	66.84 ± 59.21	71.84 ± 69.52	0.561
Preoperative LMS	12.99 ± 4.93	12.67 ± 4.33	13.32 ± 5.54	0.799
Preoperative SNOT-22	46.69 ± 18.17	51.31 ± 22.90	52.08 ± 13.45	0.397
Preoperative mLKS	3.59 ± 2.662	2.96 ± 2.207	4.07 ± 2.825	0.101
Preoperative KVSS (II)	29.63 ± 11.85	28.35 ±12.14	30.9 ± 11.55	0.766
Follow up (mo)	17.84 ± 3.85	17.60 ± 4.04	18.08 ± 3.65	0.104

Values are presented as mean ± SD. SI, saline irrigation; BI, budesonide irrigation; M, male; F, female; Y/N/U, yes/no/unknown; HTN, hypertension; ESS, endoscopic sinus surgery; CRSwNP, Chronic Rhinosinusitis with Nasal Polyps; CRSsNP, Chronic Rhinosinusitis without Nasal Polyps; ECRS, Eosinophilic Chronic Rhinosinusitis; NECRS, Non-Eosinophilic Chronic Rhinosinusitis; Eo, Eosinophil count; ECP, Eosinophil Cationic Protein; IgE, total immunoglobulin E; HPF, High-Power Field; LMS, Lund–Mackay CT score; SNOT-22, 22-item Sinonasal Outcome Test; mLKS, modified Lund–Kennedy endoscopic score; KVSS II, Korean version of the Sniffin’ Sticks test II.

## Data Availability

The data are available from the corresponding author upon reasonable request.
